# Multiple Transport-Active Binding Sites Are Available for a Single Substrate on Human P-Glycoprotein (ABCB1)

**DOI:** 10.1371/journal.pone.0082463

**Published:** 2013-12-05

**Authors:** Eduardo E. Chufan, Khyati Kapoor, Hong-May Sim, Satyakam Singh, Tanaji T. Talele, Stewart R. Durell, Suresh V. Ambudkar

**Affiliations:** 1 Laboratory of Cell Biology, Center for Cancer Research, National Cancer Institute, National Institutes of Health, Bethesda, Maryland, United States of America; 2 Department of Pharmaceutical Sciences, College of Pharmacy and Health Sciences, St. John’s University, Queens, New York, United States of America; Russian Academy of Sciences, Institute for Biological Instrumentation, Russian Federation

## Abstract

P-glycoprotein (Pgp, ABCB1) is an ATP-Binding Cassette (ABC) transporter that is associated with the development of multidrug resistance in cancer cells. Pgp transports a variety of chemically dissimilar amphipathic compounds using the energy from ATP hydrolysis. In the present study, to elucidate the binding sites on Pgp for substrates and modulators, we employed site-directed mutagenesis, cell- and membrane-based assays, molecular modeling and docking. We generated single, double and triple mutants with substitutions of the Y307, F343, Q725, F728, F978 and V982 residues at the proposed drug-binding site with cys in a cysless Pgp, and expressed them in insect and mammalian cells using a baculovirus expression system. All the mutant proteins were expressed at the cell surface to the same extent as the cysless wild-type Pgp. With substitution of three residues of the pocket (Y307, Q725 and V982) with cysteine in a cysless Pgp, QZ59S-*SSS*, cyclosporine A, tariquidar, valinomycin and FSBA lose the ability to inhibit the labeling of Pgp with a transport substrate, [^125^I]-Iodoarylazidoprazosin, indicating these drugs cannot bind at their primary binding sites. However, the drugs can modulate the ATP hydrolysis of the mutant Pgps, demonstrating that they bind at secondary sites. In addition, the transport of six fluorescent substrates in HeLa cells expressing triple mutant (Y307C/Q725C/V982C) Pgp is also not significantly altered, showing that substrates bound at secondary sites are still transported. The homology modeling of human Pgp and substrate and modulator docking studies support the biochemical and transport data. In aggregate, our results demonstrate that a large flexible pocket in the Pgp transmembrane domains is able to bind chemically diverse compounds. When residues of the primary drug-binding site are mutated, substrates and modulators bind to secondary sites on the transporter and more than one transport-active binding site is available for each substrate.

## Introduction

P-glycoprotein (Pgp, ABCB1) is a member of the ATP-Binding Cassette (ABC) transporter superfamily and plays an important role in the development of multidrug resistance (MDR) in cancer cells.  This transporter utilizes energy from ATP hydrolysis for the efflux of a variety of chemically dissimilar amphipathic compounds, including anticancer drugs [1]. Pgp is one of the most intensely studied mammalian ABC transporters. However, how it is able to recognize and transport a diverse range of compounds, cyclic peptides, xenobiotics, lipids and amphipathic anticancer agents is still not well understood [2–7].

The identification of Pgp drug-binding sites has been the goal of many studies. We have previously demonstrated that two compounds can simultaneously bind to the drug-binding pocket of human Pgp [8], which was later also reported by others [9–12]. In addition, it was proposed that Pgp contains at least two transport-active drug binding sites, one of them termed the H-site due to specificity for binding Hoechst 33342 (and also colchicine) and another the R-site, as it binds rhodamine 123 and anthracyclines [13]. A third binding site for prazosin and progesterone, with allosteric properties, has also been reported [14]. Radioligand studies indicate that Pgp contains more than two drug-binding sites [15] and competition experiments have revealed up to 7 different sites [16]. Hence, all of these reports suggest that Pgp contains multiple binding sites for many different drugs. However, data about whether each drug has single or multiple binding sites is scarce. The X-ray structures of mouse Pgp with bound inhibitors showed only one binding site for QZ59-*RRR*, while two sites are available for its stereoisomer QZ59-*SSS* (although only 1.5 molecules could be solved) [17]. Recently, Julien Marcoux et al. using mass spectrometry demonstrated that cyclosporine A (CsA) binds to two sites on Pgp [18], although the question if both sites are effective for transport remained unanswered.

In the transmembrane region, Pgp exhibits a large conical cavity in what appears to be the drug-binding pocket where the cyclic peptides QZ59-*RRR* and QZ59-*SSS* bind, as revealed in the X-ray crystal structures of mouse Pgp [17]. Although both cyclic peptides bind in the same pocket, they do so at different sites and with different stoichiometry. QZ59-*RRR* binds with 1:1 protein:ligand stoichiometry while QZ59-*SSS* binds with 1:2 stoichiometry, one molecule of QZ59-*SSS* at a deeper site (in the upper leaflet of the membrane) and the other below the position of QZ59-*RRR*, with some of the residues interacting with both cyclic peptides [17]. Is this drug binding pocket common for the large number of substrates and modulators of Pgp? In the present report, we address this question for the large molecules CsA (MW 1,203) and valinomycin (MW 1,111), and the small molecules QZ59-*SSS* (MW 547, the sulfur-analog of one of the cyclic compounds used in the crystallographic studies of mouse P-gp) [17,19], tariquidar (XR9576, MW 647) [20] and 5′-fluorosulfonylbenzoyl 5′-adenosine (FSBA, MW 490) [21] (chemical structures are shown in the Figure S1-A and chemical structures of fluorescent substrates are given in Figure S1-B). Residues in human Pgp corresponding to those interacting with both cyclic peptide inhibitors in mouse Pgp were chosen for site-directed mutagenesis investigations. Thus, selected residues were substituted with cys in a cysless background (i.e., all mutants have the seven cysteine residues of Pgp replaced with alanine) [22]. Therefore, cysless Pgp is referred to as cysless wild-type (WT) Pgp and represents the control protein. All mutants were expressed in High-Five insect cells for biochemical studies as well as in BacMam baculovirus-transduced HeLa cells for characterization of transport function. 

Upon mutation of three residues (Y307, Q725 and V982) in the pocket, some of the compounds including CsA, valinomycin and tariquidar lose their ability to inhibit the photolabeling of Pgp with [^125^I]-iodoarylazidoprazosin (IAAP), a prazosin analog transported by Pgp [23]. These observations indicate that these drugs cannot bind at their primary or natural binding site when residues of the pocket are mutated. Interestingly, the drugs can still bind Pgp mutants at other sites (here referred to as secondary sites), as reflected by ATP hydrolysis measurements showing modulation of ATPase activity upon addition of drugs, a clear indication that these compounds interact with the mutant Pgps. Furthermore, transport of fluorescent substrates in HeLa cells expressing the mutant Pgps is not significantly altered compared to cysless WT protein. Molecular modeling studies further support the selection of residues for the present investigations and provide insights into possible binding modes of large and small molecules. In summary, the residues studied in the present report are indeed part of a common drug-binding pocket, where several structurally dissimilar compounds can bind. Upon alteration of the primary binding site due to mutations, drugs can still bind to Pgp at secondary sites. Collectively these data demonstrate that each drug-substrate can bind to more than one site and all sites are capable of transport function. 

## Results

### Selection of residues of the drug-binding pocket of Pgp for mutagenesis

The X-ray structures of mouse Pgp in complex with two cyclic peptide inhibitors, QZ59-*RRR* and QZ59-*SSS*, provided support for the previously published biochemical and mutagenesis data on the drug-binding pocket of Pgp [17]. Residues that were found to interact with the substrates and inhibitors were selected for mutagenesis studies. The cysteine mutagenesis strategy has proved very useful because of the specific reactivity of cysteine residues towards thiol-reactive compounds [24]. Single cysteine mutations can be labeled (or protected from labeling) and the mutant subjected to biochemical analysis (e.g. ATPase activity) [25]. This approach has been extensively used to identify the residues of drug-binding sites. Certainly, the utility of cysteine mutagenesis studies is based on the fact that the protein is still active when all natural cysteine residues are replaced with alanine or serine, a condition fulfilled by Pgp [22,26]. Therefore, the following six residues were mutated to cysteine: Y307, F343, Q725, F728, F978 and V982; all these mutants were created in a cysless background. For biochemical studies, crude membranes were prepared from High-Five insect cells infected with baculovirus coding for cysless WT, single mutants Y307C, F343C, Q725C, F728C, F978C, V982C, double mutants Y307C/V982C, F343C/V982C, Q725C/V982C, F728C/V982C, and a triple mutant Y307C/Q725C/V982C. 

### Selected substrates and modulators do not inhibit the photo-crosslinking of mutant Pgps with IAAP

**Figure 1:**
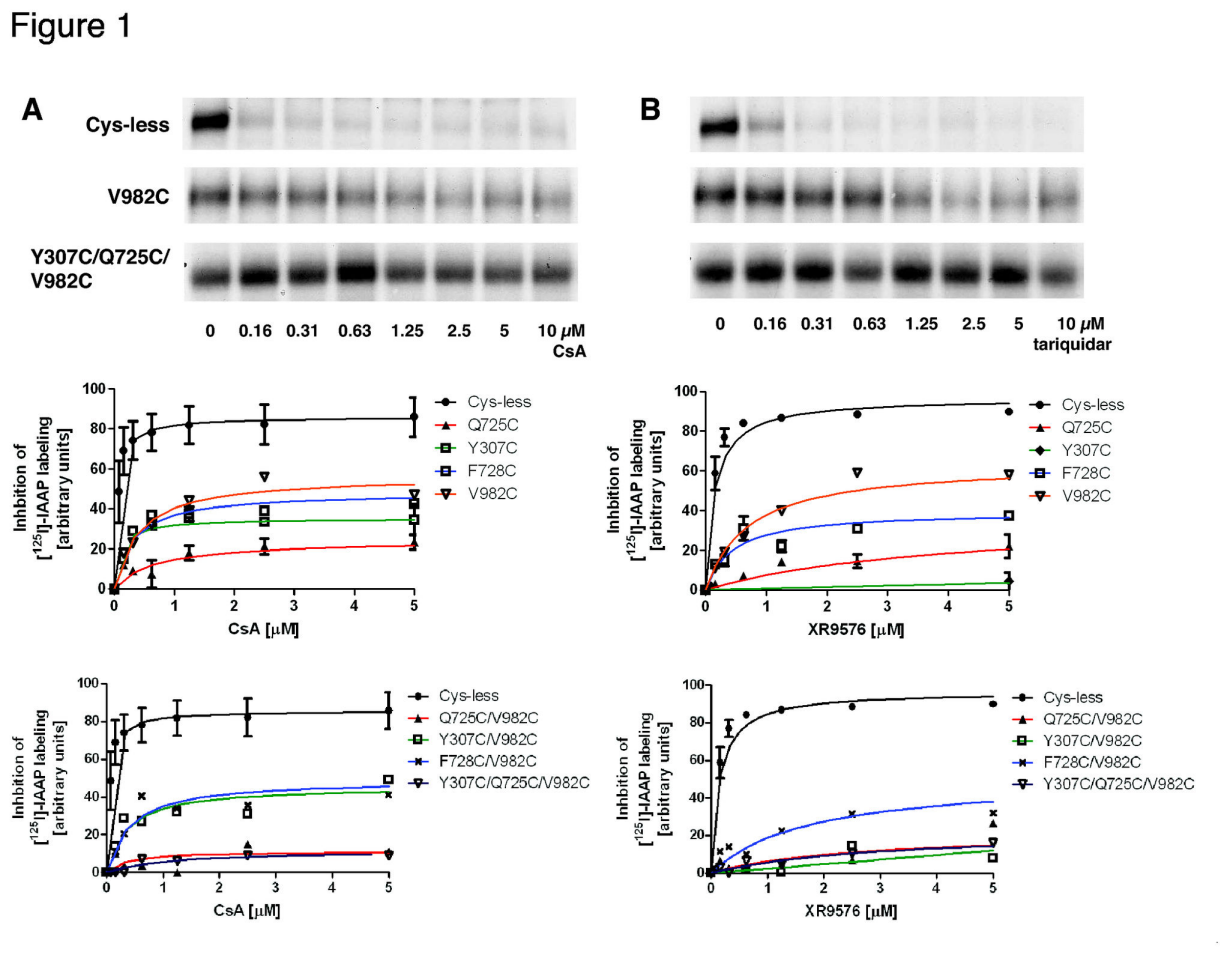
Inhibition of photocrosslinking of mutant Pgps with IAAP by cyclosporine A and tariquidar. Inhibition of IAAP labeling for single mutants Q725C, Y307C, F728C and V982C (upper graphs) and for double Q725C/V982C, Y307C/V982C, F728C/V982C, and triple Y307C/Q725C/V982C (lower graphs) mutants at different concentrations of (A) CsA and (B) tariquidar, are shown. Inhibition of IAAP labeling for cysless WT is included in all graphs, as a reference. Autoradiograms corresponding to cysless, V982C and Y307C/Q725C/V982C, as representative examples of complete inhibition, partial inhibition and no inhibition of IAAP-labeling, respectively, are shown at the top of the figure. Crude membranes containing Pgp (60-80 µg protein) were treated with increasing concentrations of CsA and tariquidar in 100 µL buffer containing 50 mM MES-Tris pH 6.8 for 10 min at 37°C. Then, samples were photocrosslinked with IAAP at 4°C as described in the Materials and Methods section. Mean values from at least three independent experiments are plotted and error bars depict SD.

Radiolabeled IAAP has been used extensively by us and others to study the interactions of substrates and inhibitors at the drug-binding site of Pgp [8,23]. IAAP binds and photo-crosslinks cysless WT Pgp and substrates (e.g., CsA) and modulators (e.g., tariquidar) inhibit this binding (Figure 1). All drugs under investigation inhibit the photo-labeling of cysless WT Pgp with IAAP in a concentration-dependent manner. With CsA, inhibition reaches a maximum of 86 ± 3% with IC_50_ = 0.05 ± 0.01 µM, while with tariquidar a maximum inhibition of 97 ± 4% is observed with IC_50_ = 0.14 ± 0.03 µM (see Figure 1 and Table 1). QZ59-*SSS*, valinomycin and FSBA inhibit IAAP-labeling up to 72%, 82% and 85% (IC_50_ = 6 µM, IC_50_ = 0.6 µM and IC_50_ = 390 µM), respectively (see Figures S2, S3, and S4). However, CsA, tariquidar and valinomycin lose almost completely this ability to inhibit IAAP photo-labeling when residues Y307, Q725 and V982 are mutated to cysteine (i.e., Y307C/Q725C/V982C mutant). QZ59-*SSS* and FSBA are able to inhibit slightly (< 30%) at higher concentrations. These observations indicate that the residues mutated to cysteine form part of the drug-binding site. The fact that drugs cannot inhibit IAAP labeling of mutant Pgps to the same extent as cysless WT Pgp, even at higher concentrations, indicates that it is unlikely they bind to the same site with lower affinity.

**Table 1:** Inhibition of IAAP-labeling of mutant Pgps with cyclosporine A and tariquidar.

**Mutation(s)**	**CsA**		**Tariquidar**	
	Max Inhibition (%)	IC_50_ (µM)	Max Inhibition (%)	IC_50_ (µM)
**Cysless WT**	86 ± 3	0.05 ± 0.01	97 ± 4	0.14 ± 0.03
**Q725C**	24 ± 4	--	37	--
**Q725C/V982C**	11	--	22	--
**Y307C**	35 ± 2	--	ND	--
**Y307C/V982C**	46	--	ND	--
**F728C**	48	--	40	--
**F728C/V982C**	49	--	ND	--
**V982C**	56	0.40	64	0.70
**Y307C/Q725C/V982C**	12	--	23	--
**F978C**	86	0.54	73	3.6

Mean values with standard errors are reported when more than two experiments were carried out; otherwise only average values are reported. IC_50_ values are reported when inhibition is higher than 50%. These values were derived from graphs similar to those shown in Figure 1.

The mutation Q725 → Cys has a major effect on CsA binding, while the mutation Y307 → Cys has a major effect on binding of tariquidar (Figure 1). For valinomycin, mutations of Q725 and V982 have a more significant effect in terms of drug binding than Y307, although it is necessary to mutate all three residues to have a complete loss of binding (Figure S3). The partial inhibition of IAAP labeling by single and double mutations suggests that modification of the drug-binding pocket, changing even a single residue, affects drug binding. When three residues are mutated, these compounds do not bind at their primary binding site where drug-binding normally blocks IAAP cross-linking.

One mutant, F978C, behaves in a manner similar to that of cysless WT Pgp, with only a change in the binding affinity for CsA, IC_50_ = 0.54 µM, compared to 0.05 µM for cysless WT Pgp (Table 1). This observation suggests that the F978 → Cys mutation is well tolerated although the lower affinity also reflects that this Phe residue takes part in the binding of CsA to Pgp. Similarly, tariquidar inhibits IAAP labeling of F978C to the same extent as cysless WT Pgp, also with a change in affinity, IC_50_ = 3.6 µM, compared to 0.14 µM for cysless WT Pgp (Table 1). However, it is quite possible that this residue may be critical for the binding of other substrates or modulators not tested here.

### A majority of mutant Pgps exhibit low basal ATPase activity, but retain their modulation by substrates and inhibitors

**Figure 2:**
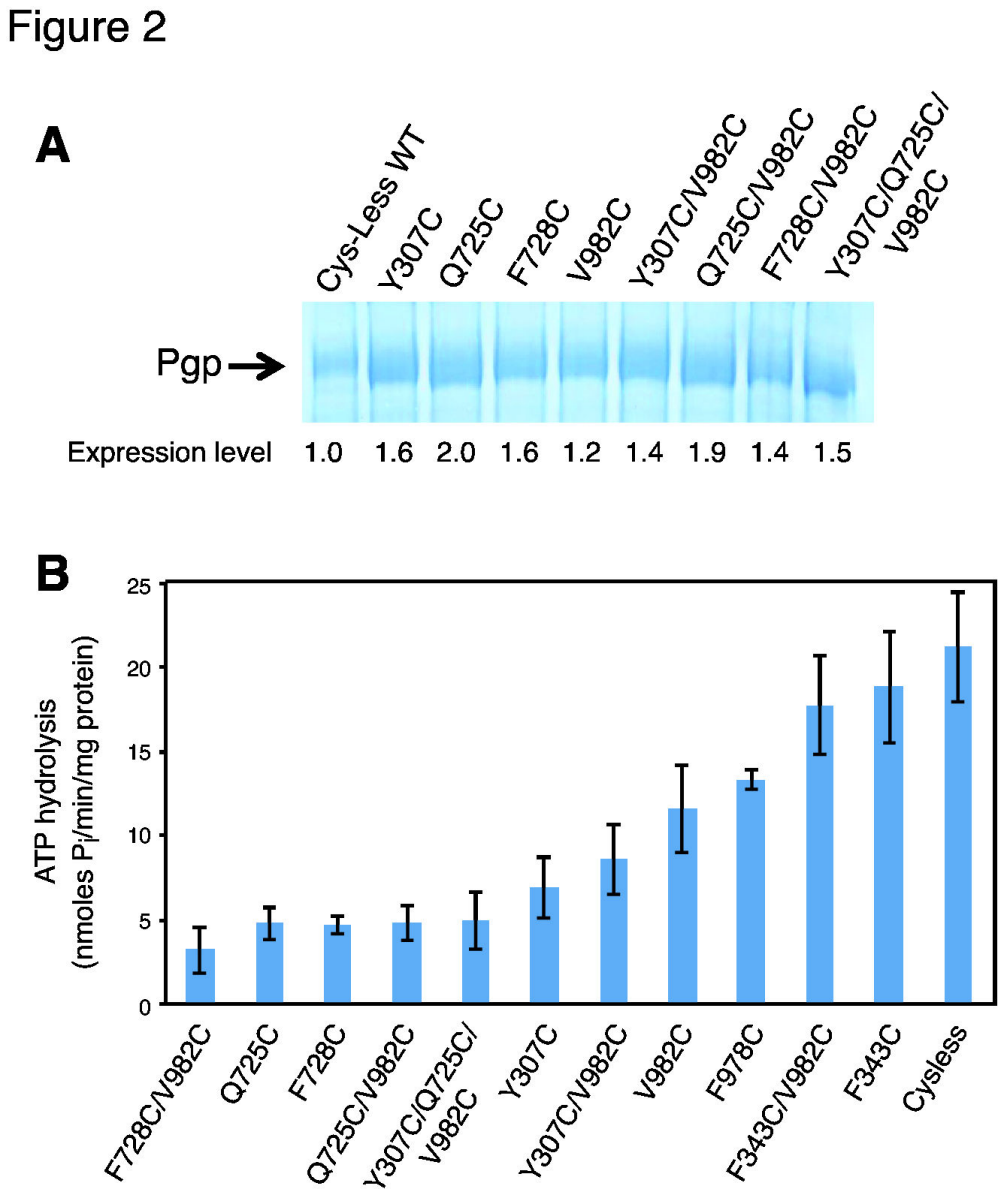
Some of the drug-binding pocket mutants exhibit very low basal ATPase activity. (A) The expression of mutant Pgps in High-Five insect cell membranes. Crude membranes of High-Five insect cells expressing indicated mutant Pgps were analyzed by standard SDS-PAGE method and proteins were stained with Colloidal Blue staining. Each lane contains 20 µg total membrane protein. Only the portion of the 7% Tris-Acetate gel with the Pgp band is shown. Expression level of mutant Pgps was quantified and compared to cysless WT Pgp using ImageJ 1.46r. (B) Basal ATP hydrolysis (in the absence of any added compound) of cysless WT and mutant Pgps is shown in bar graph format. The mutants are ordered from the lowest to the highest basal activity. Vanadate-sensitive Pgp-mediated ATP hydrolysis was measured as described in Materials and Methods. In all experiments, mutant Pgps (10-25 µg protein/100 µL) were incubated at 37°C and the ATPase activity was measured after 20 min reaction, in the presence of 5 mM ATP. At least three experiments were carried out for each mutant and error bars represent SD.

**Figure 3:**
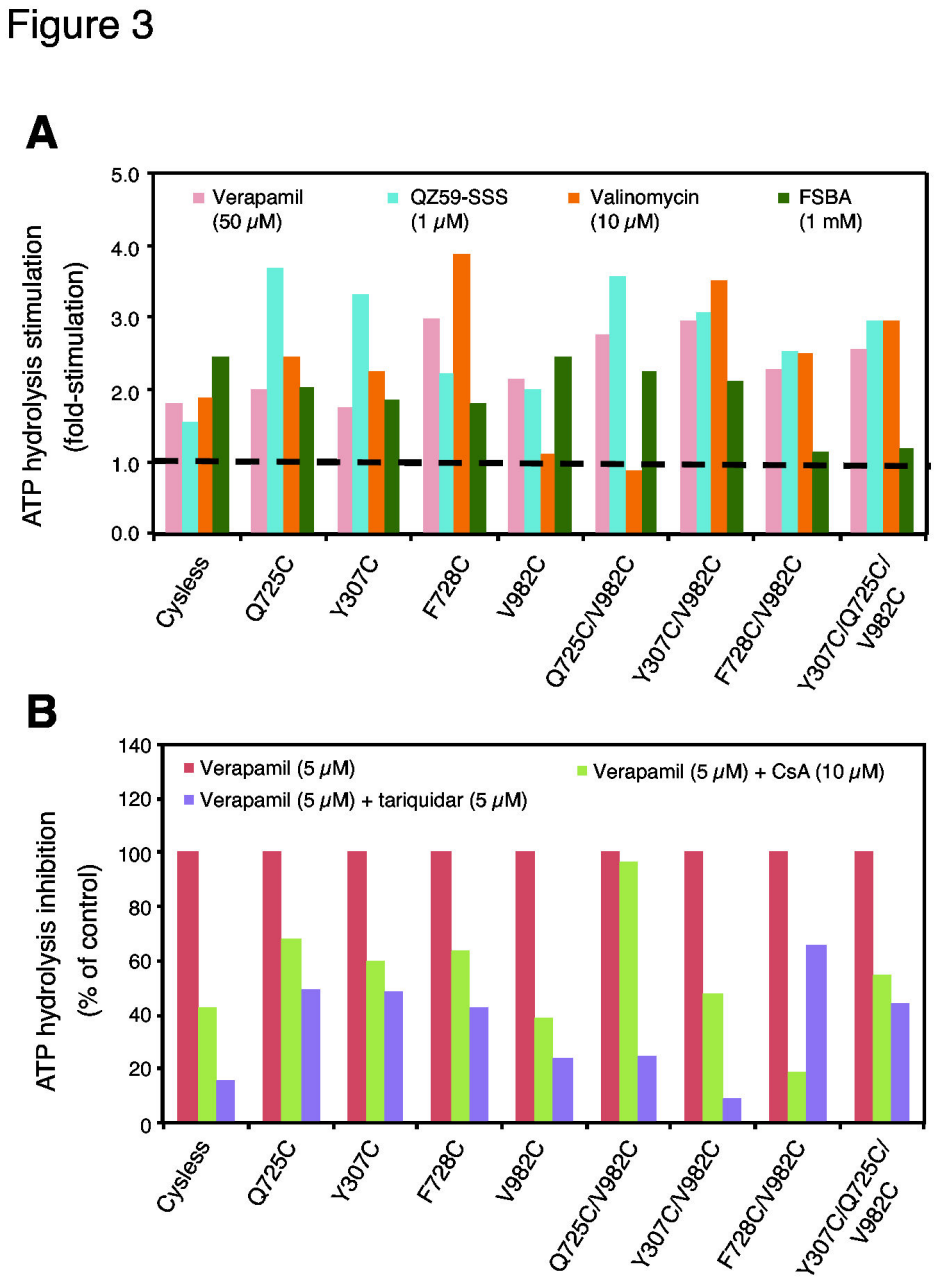
Modulation of ATP hydrolysis by drugs is retained by mutant Pgps. (A) Stimulation of ATP hydrolysis by verapamil (50 µM), QZ59-*SSS* (1 µM), valinomycin (10 µM) and FSBA (1 mM) are shown in bar graph format; basal activity (in the absence of added drug) indicated by dashed line is equal to one. (B) Inhibition of verapamil-stimulated ATP hydrolysis by CsA (10 µM) and tariquidar (5 µM) is shown. The ATPase activity is measured in the presence of verapamil (5 µM) because of the low basal activity of most of the mutants; the first bar thus indicates the activity in the presence of 5 µM verapamil, which is taken as 100% (control). Vanadate-sensitive Pgp-mediated ATP hydrolysis was measured as described in Materials and Methods. In the case of FSBA, 5 mM ATP was added prior to the addition of FSBA [21]. At least two independent experiments were carried out for each mutant with indicated compounds. Additional information including standard deviations, fold-stimulation or % inhibition, and number of experiments is given in the section (Tables S2 and S3).

As the residues in the proposed drug-binding pocket were mutated, we wanted to investigate whether the basal (in the absence of any added substrate or modulator) ATPase activity is affected by the mutation(s). Crude membranes of High-Five insect cells expressing cysless WT Pgp exhibit a basal ATPase activity of 17-25 nmol P_i_/min/mg protein, with batch-to-batch variation. The mutants F343C and F343C/V982C also exhibit normal basal activity. However a majority of the mutants show low basal activity; F728C/V982C shows the lowest (4 ±1.6 nmol P_i_/min/mg protein) while V982C and F978C shows fairly low activity (11 ± 2.2 and 13 ± 0.6, respectively). The rest of the mutants, Q725C, Y307C (and their corresponding double and triple mutants) and F728 show intermediate levels of basal ATPase activity. The expression level of the mutants in High- Five insect cell membranes is similar to or higher than that of cysless WT Pgp (as determined by the colloidal-blue stained band in SDS-PAGE gels, Figure 2A). However, the basal activity of most of the mutants is significantly lower than that of cysless WT Pgp. Figure 2B illustrates the basal ATPase activity values for all mutants under study and cysless WT Pgp. Even though the basal activity of mutants is significantly low, their ATPase activity was modulated by all substrates and modulators tested in the present investigations (see Figure 3A). Verapamil (50 µM) stimulated ATP hydrolysis of all the mutants by 2-4 fold (see Table S2 for actual values). Valinomycin also stimulated ATP hydrolysis of all mutants except V982C and Q725C/V982C. It is interesting to observe that the effect of the V982C mutation is not dominant in the rest of the double mutants and even in the triple mutant Y307C/Q725C/V982C, in which case valinomycin does stimulate ATP hydrolysis. FSBA also stimulates the ATP hydrolysis of most of the mutants, with the exception of the double F728C/V982C and the triple Y307C/Q725C/V982C mutant. QZ59-*SSS* stimulates ATP hydrolysis of the mutant Pgps with no exceptions.

Both CsA and tariquidar are potent inhibitors of the ATP hydrolysis of Pgp. Although a recent report suggests that tariquidar stimulates the activity of purified human Pgp [27]. We used tariquidar from the same commercial source (MedKoo Biosciences, Chapel Hill, NC) and found that it inhibits the ATPase activity of cysless WT. The reasons for this discrepancy are not yet known. In the case of CsA, the inhibition is not complete (57 %) because this cyclic peptide is also transported by Pgp [28]. However, tariquidar inhibits ATP hydrolysis to a greater extent (84%), reflecting the fact that tariquidar is not a substrate for Pgp [29]. The mutants exhibit low basal activity, which makes it difficult to measure the inhibition of the ATPase activity. Therefore, a sub-saturating concentration of verapamil (5 µM) was added in all experiments to increase the basal activity to a level where inhibition is more easily detected and quantified. The ATPase activity of all mutant Pgps was found to be inhibited by CsA and tariquidar (Figure 3B), showing clearly that when the residues at the drug-binding site were mutated, these inhibitors can interact with the transporter to have an effect on the ATPase activity.

### Transport of fluorescent substrates by mutant Pgps in HeLa cells

**Figure 4:**
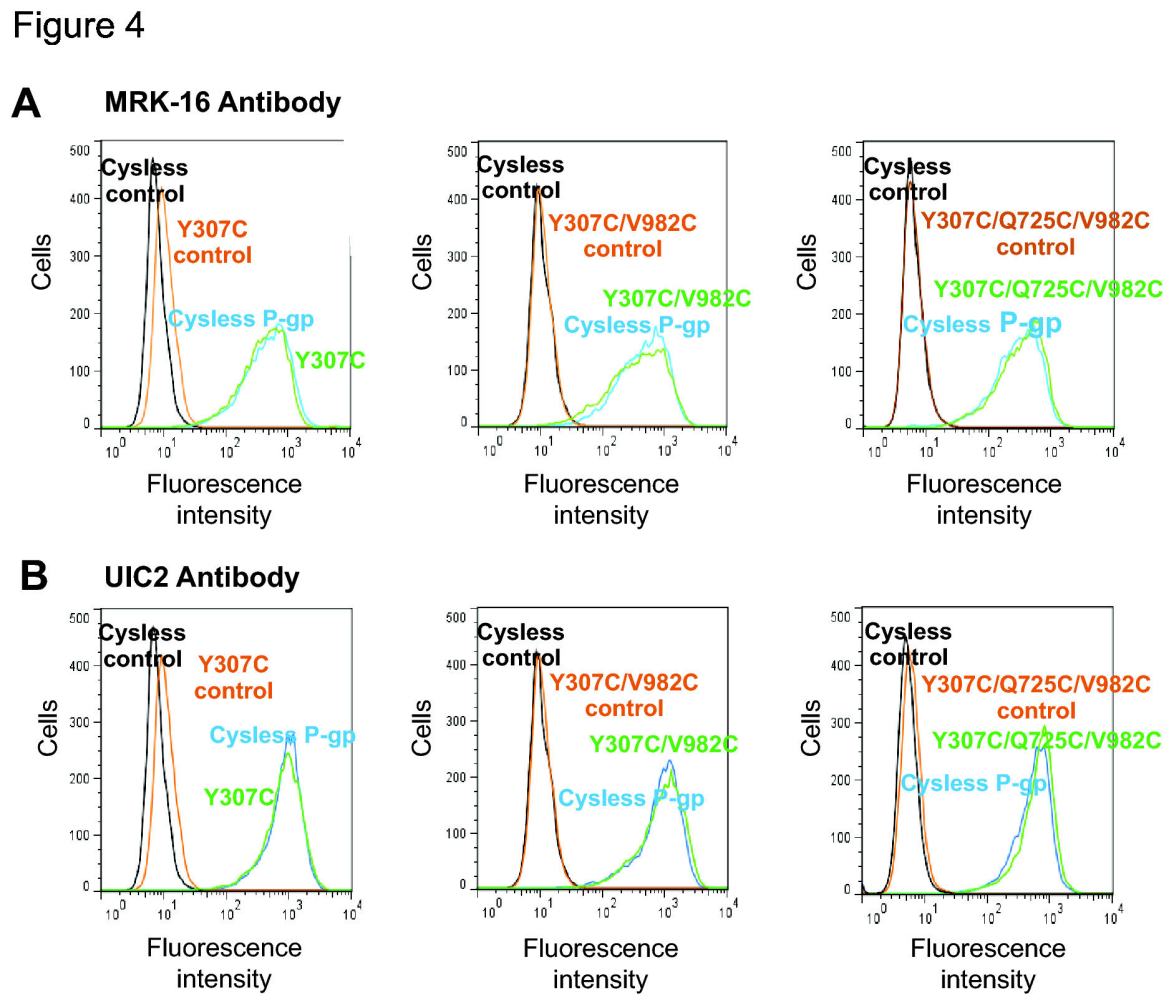
BacMam-baculovirus-transduced HeLa cells express normal levels of mutant Pgps at the cell surface. (A) The left panel shows the cell surface localization of Y307C with human Pgp-specific monoclonal antibody MRK-16 labeling as detected by green fluorescence detector. The middle panel shows the same for the double mutant Y307C/V982C, and the right panel for the triple mutant Y307C/Q725C/V982C. (B) The panels show the detection of the mutant Pgps with conformation-sensitive monoclonal UIC2 antibody after the treatment of cells with 20 µM CsA for 5 min at 37°C as described in Materials and Methods. The conformation sensitivity towards CsA of single (Y307C), double (Y307C/V982C) and triple (Y307C/Q725C/V982C) mutants was similar to cysless WT Pgp, as shown in the three panels, respectively. The histograms in both (A) and (B) are from one representative experiment, which was done independently at least three times. Samples treated with IgG2a isotype antibody are labeled as control. In all panels, the level of expression of cysless WT Pgp is shown for comparison and it is taken as 100% (see Table 2).

The results of the biochemical assays were further corroborated by intact cell assays using HeLa cells and a BacMam baculovirus expression system to characterize the transport function of mutant Pgps [30]. Cysless WT and mutant Pgps showed almost identical levels of cell surface expression, as detected by human Pgp specific MRK-16 antibody. In Figure 4A, representative histograms show the cell surface expression for single (Y307C), double (Y307C/V982C) and triple (Y307C/Q725C/V982C) mutants. The cell surface expression levels for all mutants as detected by MRK-16 antibody are summarized in Table 2. To further show that the mutant protein being expressed on the cell surface is properly folded and retains the wild-type conformation, we used UIC2, a conformation-sensitive antibody. Figure 4B shows the same levels of expression and proper folding of single, double and triple mutants as detected by UIC2 antibody using the shift assay described in Materials and Methods.

**Table 2:** The mutant Pgps expressed in HeLa cells exhibit normal cell surface expression but variable steady-state level of transport of different substrates.

**Mutation(s)**	**Cell surface expression**	**Transport function**					
		**CalAM**	**BD-PRA **	**NBD-CsA **	**Rh123**	**Dauno**	**BD-PAC**
Y307C	100	90-100	80-90	90-100	90-100	90-100	90-100
Q725C	100	90-100	90-100	90-100	90-100	90-100	90-100
F728C	100	90-100	80-90	90-100	90-100	90-100	90-100
V982C	100	90-100	80-100	<10	90-100	90-100	90-100
F343C	50-60	90-100	80-100	90-100	90-100	90-100	90-100
F978C	100	90-100	90-100	90-100	90-100	90-100	90-100
Y307C/V982C	100	90-100	50-60	50-60	90-100	90-100	90-100
Q725C/V982C	100	90-100	80-90	<20	90-100	90-100	90-100
F728C/V982C	30-40	55-65	30-40	<20	70-80	50-60	90-100
F343C/V982C	70-80	90-100	80-90	<20	90-100	90-100	90-100
Y307/Q725C/V982C	100	90-100	30-40	50-60	70-80	60-70	90-100

**Figure 5:**
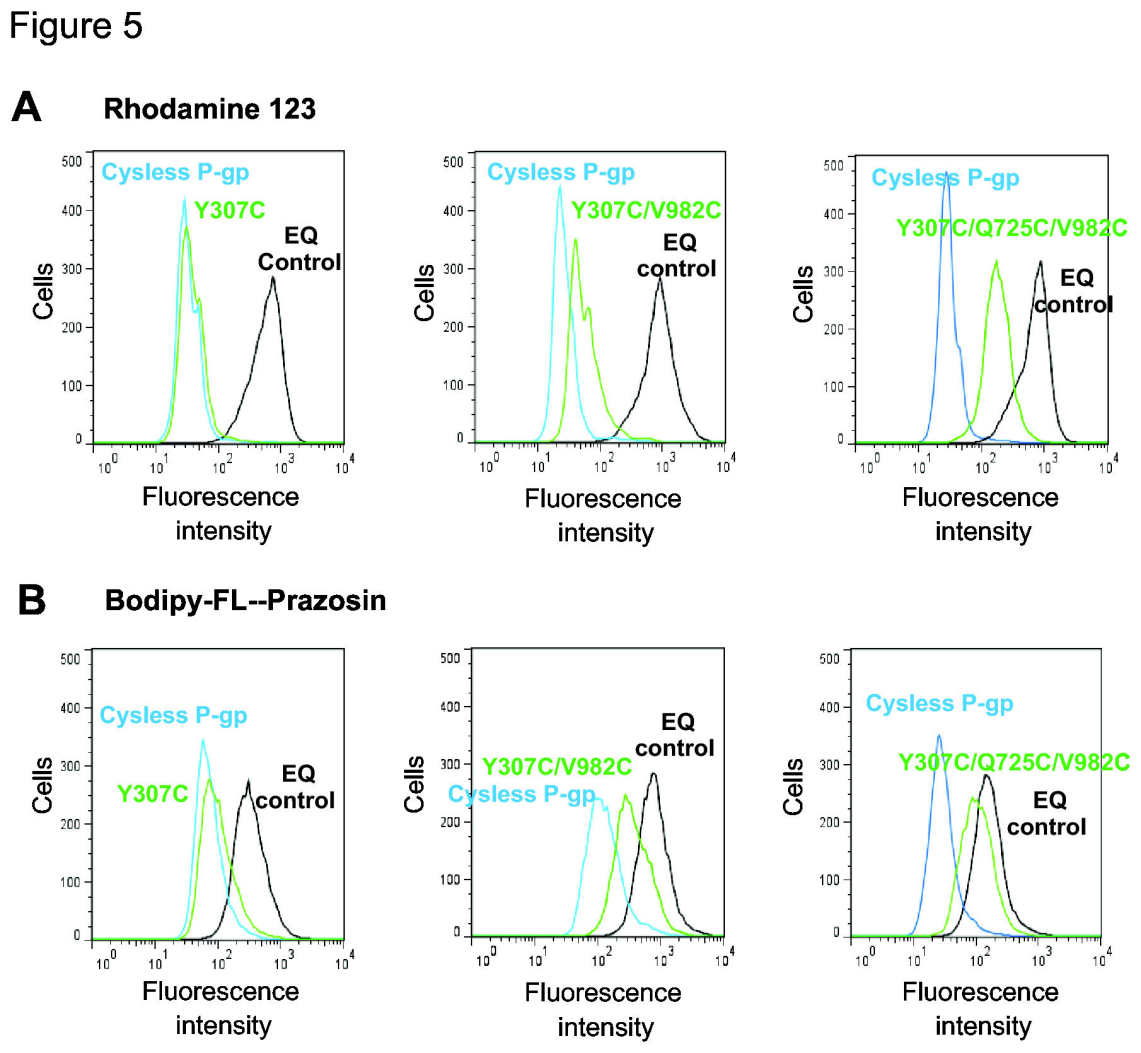
The transport functions of single (Y307C), double (Y307/V982C) and triple (Y307C/Q725C/V982C) mutants are differentially affected. (A) Rhodamine 123 and (B) Bodipy-FL-prazosin accumulation in HeLa cells. The inactive E501Q/E1201Q (EQ) mutant P-gp is used as a control to show completely aborted function as described in Materials and Methods. The HeLa cells expressing mutant-Pgps were incubated with either rhodamine 123 (1.3 µM) or bodipy-FL-prazosin (0.5 µM) for 45 min at 37°C. The cells were washed and subsequently analyzed by flow cytometry as described in Materials and Methods. The histograms show results from a typical experiment. Similar results were obtained in at least 3-4 experiments. The transport function of all mutants with six fluorescent substrates is summarized in Table 2.

For cell surface expression, the cells were incubated with MRK-16 antibody for 30 min followed by FITC-labeled anti-mouse secondary antibody for 30 min. The cells were then washed and analyzed by flow cytometer. For evaluation of function, the HeLa cells were incubated with calcein-AM for 10 min and with all other mentioned substrates for 45 min. The cells were washed and subsequently analyzed by flow cytometry. The level of cell surface expression and accumulation of substrates in HeLa cells expressing these mutant Pgps was compared to that of the cysless WT Pgp (taken as 100%). The values indicate range of expression level or steady-state accumulation of substrates from at least three independent experiments and representative histograms are shown in Figures 4 and 5. CalAM: calcein-AM; BD-PRA: bodipy-FL-prazosin; NBD-CsA: NBD-cyclosporine A; Rh123: rhodamine123; Dauno: daunorubicin and BD-PAC: bodipy-FL-paclitaxel.

HeLa cells expressing these mutant Pgps were able to efflux calcein-AM, bodipy-FL-prazosin, NBD-CsA, rodhamine 123, daunorubicin and bodipy-FL-pacitaxel. In most cases the mutants have transport function levels similar to those of cysless WT Pgp. Even with the triple (Y307C/Q725C/V982C) mutant the efflux of none of the above-mentioned substrates is completely abolished, although many of these substrates are transported at lower levels when compared to the cysless WT Pgp (Table 2). Figure 5 represents a typical histogram and shows the transport function of two substrates in the single, double and triple mutant. Figure 5A shows the transport of rhodamine 123 (Rh123), which is normal for the single (Y307C) and double (Y307C/V982C) mutants but is decreased considerably for the triple (Y307C/Q725C/V982C) mutant. Similar levels of defective function are seen for other substrates such as NBD-CsA and daunorubicin (Table 2). Notably, even three simultaneous mutations do not completely abolish Rh123 efflux. The transport of bodipy-FL-prazosin (BD-Pra) is reduced upon introduction of three mutations, although it is not totally abolished, as depicted in Figure 5B. The transport of calcein-AM and BD-paclitaxel is normal for almost all mutants, as summarized in Table 2.

To compare transport function in intact cells with binding properties in crude membranes employing the same chemical compound, NBD-CsA was used for biochemical analysis in crude membranes. NBD-CsA, similar to CsA (see above), inhibits the IAAP labeling of cysless WT Pgp in a concentration-dependent manner, with a maximum inhibition of 87% and IC_50_ = 0.05 µM (Table S1). NBD-CsA loses the ability to inhibit the IAAP labeling of the triple (Y307C/Q725C/V982C) mutant, as observed for CsA. Partial inhibition (40-50%) was observed at the highest tested concentration (10 µM) for single and double mutants (Table S1). These data show that NBD-CsA interacts with mutant Pgps in the same way as CsA.

Interestingly, the only single mutation that has a severe effect on drug transport is V982C. Cells over-expressing the V982C mutant fail to transport NBD-CsA completely. However, NBD-CsA is able to partially inhibit IAAP labeling in crude membranes (51%, Table S1), clearly showing that NBD-CsA binds the V982C mutant. Therefore, transport failure is due to a defect at a step after drug binding. As expected, the corresponding double mutants (Q725C/V982C; F728C/V982C; F343C/V982C) show dramatically reduced transport of NBD-CsA. Nonetheless, Y307C/V982C and the triple mutant Y307C/Q725C/V982C show some rescue of the NBD-CsA transport. This can most likely be attributed to the presence of the Y307C mutation, as this particular double mutant exhibits about 50-60% transport function with respect to NBD-CsA (Table 2). 

### Homology modeling of human Pgp and docking studies

The only reported three-dimensional structures of a mammalian ABC transporter with bound ligands are the X-ray structures of mouse Pgp complexed with QZ59-*RRR* and QZ59-*SSS*, published in 2009 to a resolution of 4.35-4.40 Å [17]. The mouse Pgp (mdr1a) amino acid sequence is highly similar to human Pgp (87% sequence identity and 94% similarity). Further, all residues in the binding pocket of mouse Pgp are conserved in human Pgp, with the only exception of Ser725 (Ala729 in human Pgp), which is found to interact with QZ59-*RRR* but with no apparent role for the -OH group. In other words, the pocket in mouse Pgp is essentially the same as in human Pgp, at least in terms of amino acid sequence. In 2012, the X-ray structure of *Caenorhabditis elegans* Pgp revealed some register shifts in model building of the mouse X-ray structures published in 2009 [31]. The errors in mouse Pgp model building indicate that these structures cannot be used as accurate templates for homology modeling of human Pgp.

Recently, new and corrected X-ray structures of mouse Pgp in the apo (without QZ59-*RRR* or QZ59-*SSS*-bound) conformation were published [32]. Although the resolution (3.8 Å) is still low, the accuracy of the new structures was validated by introducing 17 single-site cysteine mutations at different locations (mainly in the TMD) and labeling them with mercury(II). The identity and position of the corresponding mercury-labeled cysteine residues were determined by X-ray anomalous data [32]. Thus, the new structures confirmed that TM5 of the original structures has a register shift of one amino acid. In other words, the position that was assigned to Y303 corresponds to I302 (Y307 and I306 in the human Pgp sequence, respectively) (see Figure S5). For these reasons, a homology model of human Pgp was built based on the new corrected mouse Pgp structure (4KSB.pdb). Although the structure of Pgp from *C. elegans* was determined at higher resolution (3.4 Å) [31], the low sequence similarity to human Pgp, 46% total sequence identity and only 21% sequence identity at the drug binding site, does not make it a good template for modeling the drug-binding pocket of human Pgp.

The cysteine mutagenesis data showed that residues interacting with the QZ59-cyclic peptide inhibitors are part of the common drug-binding pocket of CsA, tariquidar, valinomycin and FSBA. To assess how these structurally different compounds bind in this pocket, we carried out docking studies at the central cavity of the homology model of human Pgp. The program AutoDock Vina [33] was used to predict possible binding modes of the ligands. All residues of the drug-binding pocket that interact with both QZ59-peptides were set as flexible for the docking jobs. The low resolution of the X-ray structure used for homology modeling (3.8 Å), in addition to the fact that drug binding is likely accomplished through an induced-fit mechanism [34] supports the use of a flexible receptor for docking studies. Dolghih and colleagues provided evidence that treating side-chains of Pgp residues as flexible is critical in order to obtain better docking scores [35].

**Figure 6:**
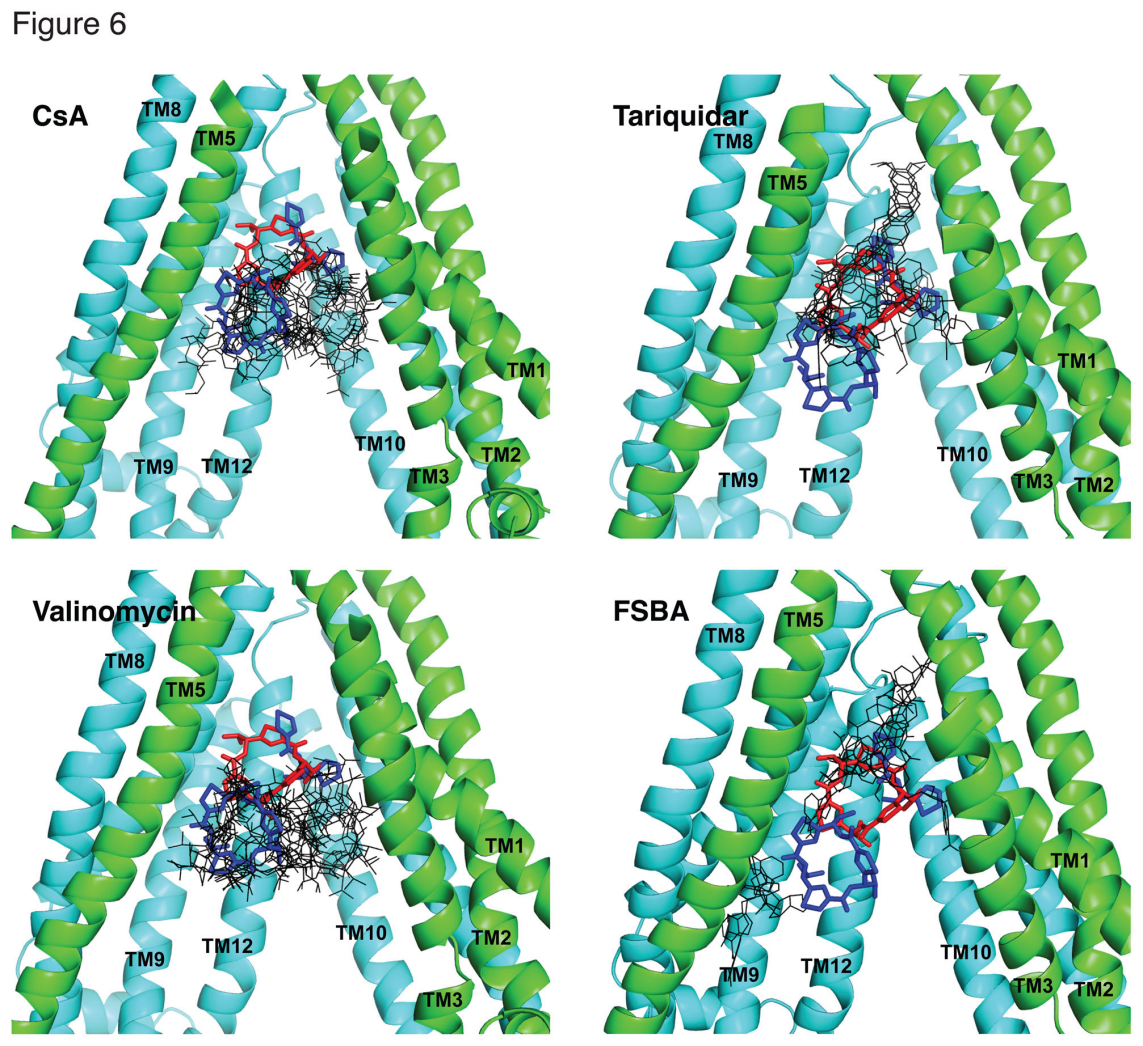
Docking of cyclosporine A, tariquidar, valinomycin and FSBA in the binding pocket of human Pgp. The homology model of human Pgp was generated using the recently described structure of mouse Pgp as a template (4KSB.pdb) [32]. Exhaustive ligand docking in the homology model of human Pgp was carried out using receptor grids centered at the position of the QZ59-*RRR* molecule (shown as red sticks), with flexible side-chains covering an ample space and a search box of dimensions 40Å × 35Å × 35Å. The first 10 modes with the highest docking scores were clustered and shown as black stick models for each drug. Docking results suggest different sites depending on the size of the molecule. QZ59-*RRR* (red) and QZ59-*SSS* (blue) molecules are included as stick models in their original positions, as indicated by the crystal structures 3G60.pdb and 3G61.pdb, respectively. Alpha-helices 4 and 5 are not shown for clarity; the rest of the helices of transmembrane domain 1 (green) and transmembrane domain 2 (cyan) are labeled and shown as cartoon models. The figure was prepared with PYMOL.

Docking studies of Pgp cannot predict the exact location of drug binding because (i) drug binding proceeds by an induced-fit mechanism, (ii) the programs cannot accurately predict the position of water molecules and (iii) the scoring functions have intrinsic limitations. However, they can indicate a number of possible poses, as shown in Figure 6. Tables with docking scores are reported in Supporting Information (Table S4). The first 10 poses have similar scores, in the range of -9.7 to -9.0 kcal/mol (CsA), -12.9 to -11.8 kcal/mol (tariquidar), -10.8 to -10.1 kcal/mol (valinomycin) and -9.9 to -9.1 kcal/mol (FSBA), indicating that all poses are possible binding locations. Contacts between the ligands and protein residues were analyzed for all poses and the interaction of each residue with the ligand (at distances shorter than 4 Å) was determined (“involvement rate [%]”) [36]. Figure S6 shows the residues that exhibit the highest involvement rate (≥50%). All the residues under investigation were found to interact at various rates with the docked molecules. Thus, docking studies provide further support for the residues selected in the present investigations as residues of the common drug-binding pocket of Pgp.

Docking studies also show that the binding sites for large and small molecules must be different. CsA and valinomycin tend to bind at the same site, where one of the molecules of QZ59-*SSS* binds mouse Pgp (lower site, extended), but also covering part of the site where QZ59-*RRR* binds Pgp. In contrast, tariquidar and FSBA tend to bind at a deeper site in the central cavity, at the position where half a molecule of QZ59-*SSS* was resolved in the crystal structure and where QZ59-*RRR* binds Pgp. It is important to note that these docking studies explored the possible binding of one molecule of ligand at the primary binding site, while the biochemical studies clearly show all drugs bind Pgp with stoichiometry higher than 1:1.

## Discussion

Pgp is known to recognize and transport a variety of chemically dissimilar compounds including amphipathic anti-cancer agents [1–3]. However, the molecular basis of the polyspecificity is not yet well understood. In addition, it is difficult to predict whether Pgp adopts the same conformation when binding any substrate or modulator, or the final structure of substrate-bound Pgp actually depends on the topology, shape and size of the substrate by an induced-fit mechanism. The observed structural flexibility of MsbA [37] and cysteine-scanning mutagenesis and oxidative cross-linking studies on human Pgp [34] certainly favors the latter mechanism. Nevertheless, the pocket where the selenazole-derivatized inhibitors bind the mouse Pgp defines a site, which we refer to as the primary binding site, and the biochemical data here reported indicate that this site is a common site for the substrates and modulators studied in the present work. 

The biochemical properties of a protein should be affected when a residue which is part of the drug-binding site is mutated. The drug should either not bind to the mutant protein or, at least, the interaction of the drug with the mutant should be different. In general terms, our mutagenesis studies suggest the latter is the case with Pgp. All drugs under investigation lose, completely or partially, the ability to inhibit the IAAP labeling of Pgp when residues Y307, Q725 and V982 together are mutated to cysteine. These observations demonstrate that the residues are part of the primary drug-binding site of Pgp for the drugs under investigation. The same residues studied in the present work were mutated to arginine by Loo and coworkers [38]; they found these mutations promote or have a neutral effect on maturation of a Pgp processing mutant (G251V) defective in folding, also confirming that these residues are in the drug translocation pathway and/or drug-binding pocket of Pgp. Previous studies have also shown that residues F728 (verapamil, cyclosporine A, vinblastine, rhodamine B) [39], V982 (verapamil, vinblastine, colchicine, rhodamine B) [25,40], and F343 (rhodamine B) [41] are part of the binding site for the drugs indicated in parentheses. 

The *C. elegans* Pgp structure revealed some register shifts in model building of TM3, TM4 and TM5 of the mouse Pgp structures (3G5U.pdb; 3G60.pdb and 3G61.pdb). The new, corrected structures (4KSB.pdb) show one amino acid shift in the helix TM5, in comparison with the original structure. The old structure has I302 outside the drug-binding pocket, while Y303 was found to interact with QZ59-*RRR*. The new structure has I302 in the drug-binding pocket, consistent with previous biochemical studies that show that this residue is part of the drug-binding site of Pgp for several drugs [42]. The new structure still has Y303 in the drug-binding pocket and the data here reported further support the involvement of Y303 (Y307 in the human sequence) in drug binding. In summary, it seems that both I302 and Y303 (I306 and Y307 in the human sequence) participate in drug binding.

Previously, drug affinity studies for activation of ATPase activity and drug-protection studies from labeling with MTS-verapamil suggested that Q725 does not directly participate in drug binding [39]. Here, we report QZ59-*SSS*, CsA, tariquidar, valinomycin and FSBA lost the ability to inhibit IAAP-labeling of Q725C to the same extent as cysless WT Pgp. These results indicate that Q725, in addition to F728, are the residues from TM7 that contribute to the drug-binding site of Pgp, also consistent with the docking studies.

The partial inhibition of IAAP labeling observed for single mutants (Y307C, Q725C, F728C, and V982C) and even double mutants (Y307C/V982C, Q725C/V982C, F728C/V982C) is indicative of some drug interaction with Pgp (Figure 1). Interestingly, the maximum inhibition (which is partial compared to cysless WT Pgp) is reached at low concentrations, suggesting that drugs might bind at secondary sites with high affinity. At these secondary sites, the IAAP labeling inhibition is approximately half of that observed when drugs bind to their primary binding sites. The fact that more than one molecule of IAAP binds and crosslinks to Pgp [8] could explain these observations. Drugs bind to cysless WT Pgp and thus block completely the photo-crosslinking of Pgp, indicating that the site where primarily drugs such as CsA bind Pgp fully alters the ability of IAAP to bind any other site on Pgp. Nevertheless, once the primary binding site is mutated, then drugs bind to secondary sites, and it seems that they are able to block half of the IAAP sites, with the other half still accessible for binding and cross-linking.

Pgp typically exhibits a basal level of ATPase activity and most of the mutants show low basal ATPase activity (see Figure 2B), which is somewhat surprising considering that this decrease in activity is produced by mutations, even by a single mutation, at the transmembrane domain, approximately 60-70 Å away from the site where ATP hydrolysis takes place. Nonetheless, the origin of the basal ATPase activity of Pgp is not known and it is a matter of debate. The behavior of these mutants is consistent with the hypothesis that basal ATPase activity is generated by interaction of membrane lipid or an endogenous substrate at the drug-binding pocket [1,43]. For this mechanism, a mutation at the site where the lipid or an endogenous substrate binds should certainly have an effect on the ATP hydrolysis.

Further support for the interpretation that drugs bind to secondary sites when their primary site is chemically modified comes from the effect of substrates and modulators on ATP hydrolysis measurements. In all mutants, even the triple Y307C/Q725C/V982C, ATP hydrolysis is inhibited by both CsA and tariquidar. In addition, valinomycin, QZ59-*SSS* and FSBA stimulate the ATP hydrolysis of the mutants. The modulation of the ATP hydrolysis by drugs is clear evidence that drugs bind to mutant Pgps, although not at the primary binding site. In other words, they bind at secondary sites.

The ability of these mutant Pgps in HeLa cells to transport at least six fluorescent substrates, even when three residues of the primary drug-binding site are mutated, add support to this interpretation. Further, these results demonstrate that secondary sites are active for transport. In steady-state conditions, calcein-AM and bodipy-placitaxel are transported by the triple (Y307C/Q725C/V982C) mutant to the same extent as cysless WT Pgp. The triple mutant is also able to transport rodhamine 123, daunorubicin, NBD-CsA and bodipy-FL-prazosin, although with less efficiency than cysless WT Pgp. Direct comparisons between biochemical data obtained with crude membranes and transport data from experiments with HeLa cells were carried out for NBD-CsA. NBD-CsA proved to have the same effect as CsA on the inhibition of IAAP labeling of Pgp and it also lost that ability in the case of the triple mutant. Therefore, NBD-CsA does not bind to its primary site on Y307C/Q725C/V982C, but to a secondary site, where it is transported at 50-60% of the rate of cysless WT Pgp.

The results of the present investigations reveal that a substrate has more than one binding site on human Pgp and each site is capable of transport function. Pgp is able to bind drugs at secondary sites when their primary binding sites are chemically modified. In view of the number of investigations that use site-directed mutagenesis of Pgp and other ABC transporters, the fact that Pgp has multiple sites for each drug should be taken into consideration when interpreting the data.

The ability of Pgp to bind to a molecule even after chemical modification of the primary binding site reveals important information concerning the molecular topology of this transporter. The central cavity holds several similar sites for drug binding, thus guaranteeing that binding is effective and transport takes place despite mutations at the primary drug-binding site. This is possible because there are several sets of essential residues for binding, in a specific 3-D arrangement [44]. The existence of several similar sites generates multiple possibilities including molecular (or chemical) flexibility that can also explain the broad substrate specificity of Pgp. The structural flexibility certainly facilitates fine tuning between the substrate and the binding site (primary or secondary) for effective binding and also helps to accommodate different molecules on multiple sites (polyspecificity). Thus, our results demonstrate that the existence of multiple binding sites for each drug makes Pgp a very efficient transporter.

## Materials and Methods

### Chemicals

[^125^I]IodoArylAzidoPrazosin (IAAP) (2200 Ci/mmol) was purchased from PerkinElmer Life Sciences (Boston, MA). Chemical synthesis of cyclic peptide QZ59-*SSS*-sulfur will be described elsewhere. Cyclosporine A was purchased from Alexis Corporation (Switzerland). Calcein-AM, bodipy-FL-prazosin and Bodipy-Paclitaxel were purchased from Invitrogen (Carlsbad, CA). Tariquidar (XR9576) was kindly provided by Dr. Susan Bates (National Cancer Institute, National Institutes of Health). NBD-cyclosporine A was a generous gift from Drs. Anika Hartz and Björn Bauer, University of Minnesota (Duluth, MN). ATP, FSBA, valinomycin and all other chemicals were obtained from Sigma-Aldrich Chemical Co. (St. Louis, MO). The Pgp-specific monoclonal antibody C219 was obtained from Fujirebio Diagnostic Inc. (Malvern, PA). MRK16 antibody was purchased from Kyowa Medex Company, Tokyo, Japan and UIC2 antibody from eBioscience (San Diego, CA).

### Cell lines and culture conditions

HeLa cells were cultured in DMEM media supplemented with 10% FBS, 1% glutamine, and 1% penicillin as described previously [27].

### BacMam baculovirus transduction of HeLa cells

The BacMam-Pgp virus (encoding cysless WT and the mutant Pgps) was added to 2.5 million HeLa cells at a titer of 50-60 viral particles per cell in 3 ml of DMEM and incubated at 37°C [30]. After 1 hr DMEM was added to make a total of 20 ml in the flask and these infected cells were further incubated for 3-4 hrs. 10 mM butyric acid was then added and the cells continued to incubate at 37°C. After 24 hrs, the cells were trypsinized, washed, counted and analyzed by flow cytometry for cell surface expression and the function of the wildtype and mutant Pgps.

Cell surface expression of cysless WT and mutant Pgps was examined with MRK16 antibody as described earlier [45,46]. Briefly, Pgp-expressing cells (250,000 cells) were incubated with MRK16 antibody (1 µg per 100,000 cells) for 60 min. Cells were subsequently washed and incubated with FITC-labeled anti-mouse secondary antibody IgG2a (1 µg per 100,000 cells; BD Biosciences, San Jose, CA) for 30 min at 37°C. The cells were washed with cold PBS and analyzed by flow cytometry using a FACSort instrument equipped with a 488 nm argon laser and 530 nm bandpass filter.

### Transport of fluorescent substrates

The transport function of cysless WT and mutant Pgps was determined using fluorescent compounds with flow cytometry as previously described [30,47,48]. Briefly, cells were trypsinized and incubated with fluorescent substrates such as calcein-AM (0.5 µM) for 10 min or rhodamine123 (1.3 µM), NBD-CsA (0.5 µM), daunorubicin (0.5 µM), bodipy-FL-prazosin (0.5 µM), bodipy-paclitaxel (0.5 µM) and bodipy-vinblastine (0.5 µM) for 45 min. Cells were washed with cold PBS and resuspended in PBS containing 0.1% BSA before analysis. The accumulation of substrates was calculated with respect to the accumulation in E556Q/E1201Q mutant Pgp, which is completely inactive [48], and is treated as a negative control for all transport experiments.

### UIC2 shift assay

The UIC2 shift assay was performed under physiologic conditions as described earlier (Mechetner et al., 1992). Briefly, 250,000 cells were resuspended in 0.5 ml of IMDM in both sample and control tubes and allowed to equilibrate at 37°C in a water bath for 5 min with cyclosporine A (20 µM) or DMSO, respectively. UIC2 antibody (1 µg per 100,000 cells) was then added to this suspension and the tubes were incubated at 37°C for another 30 min. Cells were then washed with excess IMDM, resuspended in 250 µl of IMDM and incubated with FITC-labeled anti-mouse secondary antibody (1 µg per 100,000 cells; BD Biosciences, San Jose, CA) for 30 min at 37°C. Cells were then washed and fluorescence of FITC was analyzed by flow cytometry as described above. The UIC2 shift was defined as the difference between UIC2 binding in the presence and in the absence of cyclosporine A. The mutant Pgps were compared with the cysless WT.

### Preparation of crude membranes from High-Five insect cells

High-Five insect cells (Invitrogen, Carlsbad, CA) were infected with recombinant baculovirus carrying the human *MDR*1 cDNA cysless WT and various mutant Pgps, with a His_6_-tag at the C-terminus as described previously [49]. Crude membranes were prepared as described previously [28,50]

### Photo-crosslinking of cysless WT and mutant Pgps with [^125^I]-Iodoarylazidprazosin

The crude membranes of High-Five insect cells containing 60-80 µg protein per 100 µL in 50 mM MES-Tris buffer (pH 6.8), were incubated for 10 minutes at 37°C in the absence (control) and presence of compound(s) at indicated concentration. Then the samples were incubated at 4°C under subdued light for the addition of 4-7 nM of IAAP and they were subsequently photo-crosslinked for 5 minutes with a 365 nm UV-lamp, followed by electrophoresis and quantification as described previously [50].

### ATP hydrolysis measurements

Crude membranes (10-25 µg protein/100 µ L) from High Five insect cells expressing cysless WT or mutant Pgp were incubated in the presence and absence of sodium orthovanadate (0.3 mM), in a buffer containing 50 mM MES-Tris pH 6.8, 50 mM KCl, 5 mM NaN_3_, 1 mM EGTA, 1 mM ouabain, 2 mM DTT, and 10 mM MgCl_2_. Basal activity was measured in the absence of any drug and drug-modulated activity was measured in the presence of different drugs (CsA, tariquidar, valinomycin, verapamil, QZ59-*SSS*) added as a small aliquot of dimethyl sulfoxide (DMSO) solution (final concentration of DMSO is 1%). The reaction was initiated by addition of 5 mM ATP and continued for 20 min at 37°C. In the case of the FSBA-stimulated ATPase activity, 5 mM ATP was added prior to the addition of FSBA, as previously described [21]. The reaction was stopped by addition of 100 µ L of 5% SDS and the amount of inorganic phosphate generated was quantified with a colorimetric reaction, as previously described [51].

### Computational methods

#### Homology Modeling

A homology model of human Pgp in an *open* (inward-facing) conformation was built on the basis of the recently published and corrected X-ray structure of mouse Pgp (4KSB.pdb) [32]. The sequence alignment was done by BLAST [52], and the model was constructed by *in-silico* mutation of the protein residues to the human Pgp sequence. Finally, side-chain conformations of the new residues were visually inspected and modified when necessary. The stereochemical quality of the models was inspected by PROCHECK [53] at the validation server of the Protein Data Bank; some fragments of the structures were submitted to geometrical regularization using Coot [54] to improve the stereochemical quality. The final model has no close contacts and the Ramachandran plot showed 91.68% of all residues in favored regions while 99.92% of all residues were in allowed regions (MolProbity) [55]. 

#### Docking studies

The structure (pdb files) and topology files of the ligands were prepared by crafting coupled topology (.rtf) and parameter files (.prm).  The covalent geometry and the potential energy functions representing all the bonds, angles, dihedrals and improper dihedrals were defined primarily using the 2D-sketcher and 3D-builder modules of Quanta2008 (Accelrys Software Inc.).  Finally, the energy-minimized structures of the ligands were produced using CHARMm [56] iteratively refining the topology and parameter files. The docking studies were performed using AutoDock Vina [33] and the receptor and ligand structures were prepared with the MGLtools software package [57]. Flexible receptor docking was executed for cyclosporine A, tariquidar, valinomycin and FSBA. The following residues were found to interact with the ligands in the X-ray structures of mouse Pgp in complex with the cyclic peptides QZ59-*RRR* and QZ59-*SSS*: L65, M68, M69, F72, Q195, F303, I306, Y307, F336, L339, I340, F343, Q347, N721, Q725, F728, F732, M949, Y953, F957, L975, F978, V982, M986, Q990, and S993. Therefore these residues were selected to use as flexible side–chains in the docking studies. It is important to note that F303 (F299 in mouse) and I306 (I302 in mouse) do not interact with the QZ59-peptides in the X-ray structures but are included as flexible residues because of the TM5 one amino-acid registry shift detected; for the same reason L304 (L300 in mouse) that was found to interact with QZ59-*SSS* is excluded as a flexible residue. 

A receptor grid centered at the position of the bound inhibitor QZ59-*RRR* (x=19.317, y=52.588 and z=-0.676) and an inner box of dimensions of 40Å x 35Å x 35Å were used to search for binding poses for the ligands. The exhaustiveness level was set to 40 for all jobs, which is 5 times higher than the default value (8), to reduce the probability of not finding the global minimum of the scoring function, considering the relatively large search box and the elevated number of flexible residues.

## Supporting Information

Figure S1 **Figure S1-A.** **Chemical structures of substrates and modulators used in this work**. Figure S1-B. Chemical structures of fluorescent substrates used to measure transport function of mutant Pgps.
(TIF)

Figure S2 **Effect of QZ59-*SSS*-sulfur on the photo-crosslinking of cysless WT and mutant Pgpswith IAAP.** Inhibition of IAAP-labeling for cysless WT and for triple mutant Y307C/Q725C/V982C at different concentrations of QZ59-*SSS*-sulfur are shown (graph). IAAP-labeling was carried out as described in the legend of Figure 1. Table shows the inhibition of IAAP-labeling in the presence of 15 µM QZ59S-*SSS*. Two experiments were carried out for all mutant/drug combinations and average values are reported.
(TIF)

Figure S3 **Effect of valinomycin on the photocrosslinking of cysless WT and mutant Pgps withIAAP.** Inhibition of IAAP-labeling for single mutants Q725C, Y307C and V982C (upper graph) and for double (Q725C/V982C and Y307C/V982C) and triple (Y307C/Q725C/V982C) mutants (lower graph) at different concentrations of valinomycin are shown. Inhibition of IAAP-labeling of cysless WT is included in both graphs, as a reference. Table summarizes the maximum inhibition of IAAP-labeling of mutant Pgps with valinomycin. IC_50_ values are reported when inhibition of IAAP-labeling is higher than 50%. Two independent experiments were carried out for all mutant/drug combinations and average values are reported.
(TIF)

Figure S4 **Effect of FSBA on the photocrosslinking of cysless WT and mutant Pgps with IAAP.** Inhibition of IAAP-labeling of cysless WT and triple (Y307C/Q725C/V982C) mutant at different concentrations of FSBA are shown (graph). IAAP-labeling was carried out as described in the legend of Figure 1. Table shows the inhibition of IAAP-labeling of cysless WT and mutant Pgps with 1mM FSBA. Two experiments were carried out and average values are reported.
(TIF)

Figure S5 **TM5 of original mouse Pgp X-ray structures (2009) has a register shift of one amino acid that occurred during model building.** TM5 and TM6 of X-ray structures in apo conformation of mouse Pgp 3G5U.pdb reported in 2009 [17] and recently improved 4KSB.pdb [32] were aligned with PyMOL. TM6 was removed for clarity and TM5 is shown as a cartoon model in green (3G5U.pdb) and blue (4KSB.pdb). Residues I302 and Y303 are also shown as stick models. The alignment shows a one amino-acid shift between the residues of both TM5s. In other words, the position where Y303 (Y307 in human Pgp) was assigned corresponds to I302 (I306 in human Pgp).
(TIF)

Figure S6 **Protein-ligand interactions indicated by docking poses.** Cyclosporine A, tariquidar, valinomycin and FSBA were docked at the drug-binding pocket of Pgp using a flexible receptor. The first 10 poses with the highest scores (see Table S4 and Figure 6) were analyzed for their interaction with the transporter residues. An interaction is assumed to exist when the side-chain of the ligand is at a distance shorter than 4 Å from the residue in Pgp.
(TIF)

Table S1 **Effect of NBD-cyclosporine A on IAAP labeling of selected mutant Pgps.** Effect of 0 to 5 µM concentrations of NBD-CsA and CsA on IAAP labeling of cysless WT and single, double and triple mutant Pgps was determined as described in the legend of Figure 1. IC_50_ values are reported when inhibition was higher than 50%. Standard deviations are shown when data from three or more independent experiments were available.
(DOC)

Table S2 **Basal and stimulated ATPase activity of mutant Pgps.** Basal and verapamil (50 µM), QZ59-SSS (1 µM), valinomycin (10 µM) and FSBA (1 mM) -stimulated ATPase activity of mutant Pgps are reported. Vanadate-sensitive Pgp-mediated ATP hydrolysis was measured as described in the methods section. At least two experiments were carried out for each mutant and indicated compound, and standard deviations are shown when more than two experiments were performed.
(DOC)

Table S3 **Effect of cyclosporine A and tariquidar on verapamil-stimulated ATPase activity of mutant Pgps.** Inhibition of verapamil-stimulated ATP hydrolysis by CsA (10 µM) and tariquidar (5 µM) are shown. The ATPase activity is measured in the presence of verapamil (5 µM) because of the low basal activity of most of the mutants. ver, verapamil.
(DOC)

Tables S4 **AutoDock Vina docking of cyclosporine A, tariquidar, valinomycin and FSBA in the homology model of human Pgp.** Predicted modes with their respective docking scores are reported. A flexible receptor grid centered at the position of the bound-inhibitor QZ59-*RRR* (x=19.317, y=52.588 and z=-0.676) and an inner box of dimensions 40Å x 35Å x 35Å, was used to search binding poses of the ligands. The flexible receptor was defined with 26 residues of the binding pocket (see methods section).
(DOC)
