# Supplementary figures and images for: Multiple Transport-Active Binding Sites Are Available for a Single Substrate on Human P-Glycoprotein (ABCB1)
Source: PLoS One. 2013 Dec 5;8(12):e82463. doi: 10.1371/journal.pone.0082463 (PMC3857843; doi:10.1371/journal.pone.0082463)

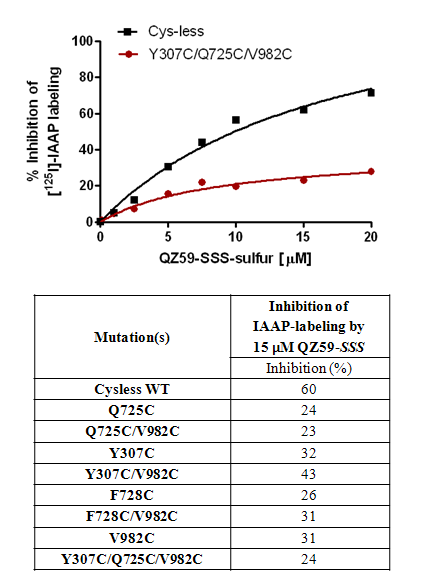

Supplement: Figure S2 — Effect of QZ59-SSS-sulfur on the photo-crosslinking of cysless WT and mutant Pgpswith IAAP. Inhibition of IAAP-labeling for cysless WT and for triple mutant Y307C/Q725C/V982C at different concentrations of QZ59-SSS-sulfur are shown (graph). IAAP-labeling was carried out as described in the legend of Figure 1. Table shows the inhibition of IAAP-labeling in the presence of 15 µM QZ59S-SSS. Two experiments were carried out for all mutant/drug combinations and average values are reported. (TIF) [file pone.0082463.s002.tif]

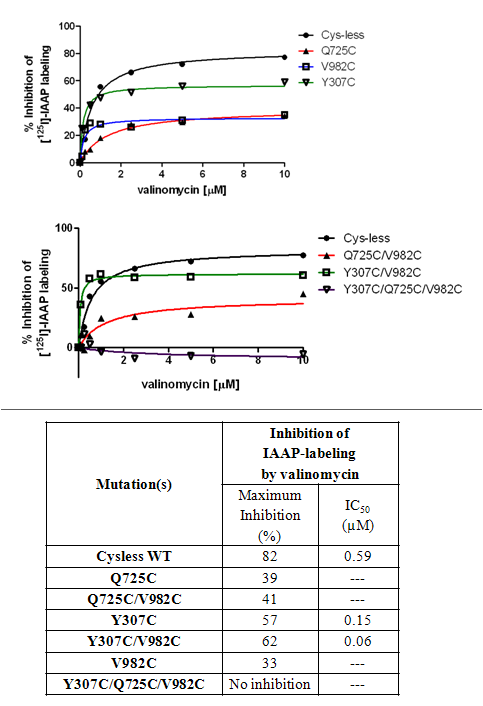

Supplement: Figure S3 — Effect of valinomycin on the photocrosslinking of cysless WT and mutant Pgps withIAAP. Inhibition of IAAP-labeling for single mutants Q725C, Y307C and V982C (upper graph) and for double (Q725C/V982C and Y307C/V982C) and triple (Y307C/Q725C/V982C) mutants (lower graph) at different concentrations of valinomycin are shown. Inhibition of IAAP-labeling of cysless WT is included in both graphs, as a reference. Table summarizes the maximum inhibition of IAAP-labeling of mutant Pgps with valinomycin. IC50 values are reported when inhibition of IAAP-labeling is higher than 50%. Two independent experiments were carried out for all mutant/drug combinations and average values are reported. (TIF) [file pone.0082463.s003.tif]

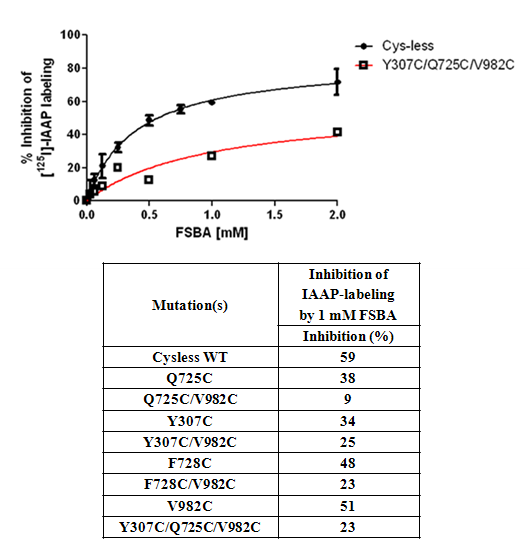

Supplement: Figure S4 — Effect of FSBA on the photocrosslinking of cysless WT and mutant Pgps with IAAP. Inhibition of IAAP-labeling of cysless WT and triple (Y307C/Q725C/V982C) mutant at different concentrations of FSBA are shown (graph). IAAP-labeling was carried out as described in the legend of Figure 1. Table shows the inhibition of IAAP-labeling of cysless WT and mutant Pgps with 1mM FSBA. Two experiments were carried out and average values are reported. (TIF) [file pone.0082463.s004.tif]

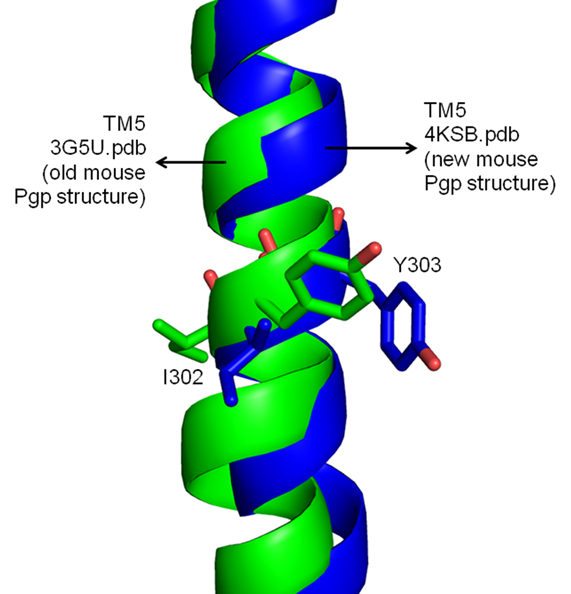

Supplement: Figure S5 — TM5 of original mouse Pgp X-ray structures (2009) has a register shift of one amino acid that occurred during model building. TM5 and TM6 of X-ray structures in apo conformation of mouse Pgp 3G5U.pdb reported in 2009 [17] and recently improved 4KSB.pdb [32] were aligned with PyMOL. TM6 was removed for clarity and TM5 is shown as a cartoon model in green (3G5U.pdb) and blue (4KSB.pdb). Residues I302 and Y303 are also shown as stick models. The alignment shows a one amino-acid shift between the residues of both TM5s. In other words, the position where Y303 (Y307 in human Pgp) was assigned corresponds to I302 (I306 in human Pgp). (TIF) [file pone.0082463.s005.tif]

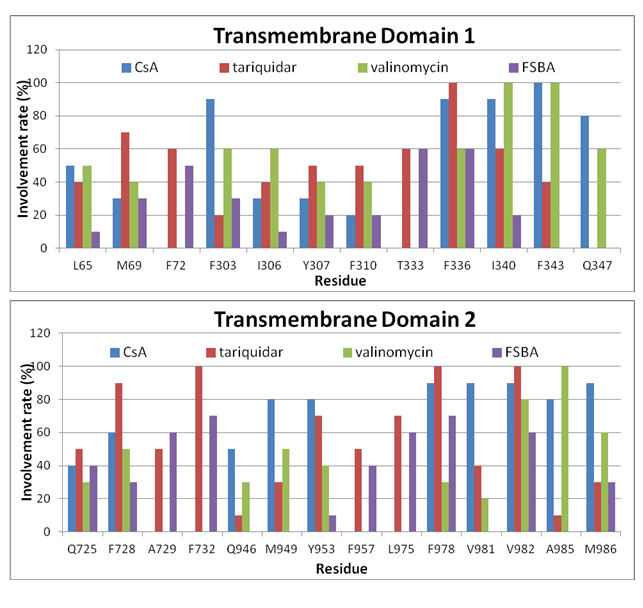

Supplement: Figure S6 — Protein-ligand interactions indicated by docking poses. Cyclosporine A, tariquidar, valinomycin and FSBA were docked at the drug-binding pocket of Pgp using a flexible receptor. The first 10 poses with the highest scores (see Table S4 and Figure 6) were analyzed for their interaction with the transporter residues. An interaction is assumed to exist when the side-chain of the ligand is at a distance shorter than 4 Å from the residue in Pgp. (TIF) [file pone.0082463.s006.tif]
