# Supplementary material for: Multiple Transport-Active Binding Sites Are Available for a Single Substrate on Human P-Glycoprotein (ABCB1)
Source: PLoS One. 2013 Dec 5;8(12):e82463. doi: 10.1371/journal.pone.0082463 (PMC3857843; doi:10.1371/journal.pone.0082463)
Supplement: Table S1 — Effect of NBD-cyclosporine A on IAAP labeling of selected mutant Pgps. Effect of 0 to 5 µM concentrations of NBD-CsA and CsA on IAAP labeling of cysless WT and single, double and triple mutant Pgps was determined as described in the legend of Figure 1. IC50 values are reported when inhibition was higher than 50%. Standard deviations are shown when data from three or more independent experiments were available. (DOC) [file pone.0082463.s007.doc]

| **Table S1.** **Effect of NBD-cyclosporine A on IAAP labeling of selected mutant Pgps** | | | | |
| --- | --- | --- | --- | --- |
| **Mutation(s)** | **Inhibition of**  **IAAP-labeling** | | | |
|  | **NBD-CsA** | | **CsA** | |
|  | Maximum  Inhibition (%) | IC_50_  (µM) | Maximum  Inhibition (%) | IC_50_  (µM) |
| **Cysless WT** | 87 | 0.05 | 86 ± 3 | 0.05 |
| **Q725C** | 38 | -- | 24 ± 4 | -- |
| **V982C** | 51 | -- | 56 | 0.40 |
| **Q725C/V982C** | 41 | -- | 11 | -- |
| **Y307C/Q725C/V982C** | 7 | --- | 12 | -- |
