# Supplementary material for: Multiple Transport-Active Binding Sites Are Available for a Single Substrate on Human P-Glycoprotein (ABCB1)
Source: PLoS One. 2013 Dec 5;8(12):e82463. doi: 10.1371/journal.pone.0082463 (PMC3857843; doi:10.1371/journal.pone.0082463)
Supplement: Table S2 — Basal and stimulated ATPase activity of mutant Pgps. Basal and verapamil (50 µM), QZ59-SSS (1 µM), valinomycin (10 µM) and FSBA (1 mM) -stimulated ATPase activity of mutant Pgps are reported. Vanadate-sensitive Pgp-mediated ATP hydrolysis was measured as described in the methods section. At least two experiments were carried out for each mutant and indicated compound, and standard deviations are shown when more than two experiments were performed. (DOC) [file pone.0082463.s008.doc]

| **Table S2. Basal and stimulated ATPase activity of mutant Pgps** | | | | | |
| --- | --- | --- | --- | --- | --- |
| Pgp mutant | DMSO/compound | ATP hydrolysis  (nmoles P_i_/min/mg protein) | | | |
|  |  | Average | Fold-stimulation | STDEV | # expts |
| Cysless WT | Basal | 21 | 1.0 | 3.7 | 13 |
| (control) | Verapamil 50 µM | 38 | 1.8 | 11.4 | 4 |
|  | QZ59-*SSS* 1 µM | 33 | 1.6 | 0.5 | 4 |
|  | Valinomycin 10 µM | 40 | 1.9 | 4.9 | 4 |
|  | FSBA 1 mM | 52 | 2.4 | 1.5 | 3 |
| Q725C | Basal | 5 | 1.0 | 1.0 | 16 |
|  | Verapamil 50 µM | 10 | 2.0 | 3.2 | 10 |
|  | QZ59-*SSS* 1 µM | 18 | 3.6 |  | 2 |
|  | Valinomycin 10 µM | 12 | 2.5 | 3.8 | 6 |
|  | FSBA 1 mM | 10 | 2.0 |  | 2 |
| Q725A | Basal | 5 | 1.0 | 1.1 | 4 |
|  | Verapamil 50 µM | 9 | 1.8 |  | 1 |
|  | QZ59-*SSS* 1 µM | 14 | 2.8 |  | 2 |
|  | Valinomycin 10 µM | 11 | 2.1 |  | 2 |
| Q725C/V982C | Basal | 5 |  | 1.6 | 12 |
|  | Verapamil 50 µM | 15 | 2.8 | 2.1 | 3 |
|  | QZ59-*SSS* 1 µM | 19 | 3.8 |  | 2 |
|  | Valinomycin 10 µM | 5 | --- |  | 2 |
|  | FSBA 1 mM | 12 | 2.3 |  | 2 |
| Y307C | Basal | 8 | 1.0 | 1.7 | 13 |
|  | Verapamil 50 µM | 13 | 1.8 | 3.4 | 5 |
|  | QZ59-*SSS* 1 µM | 25 | 3.1 |  | 2 |
|  | Valinomycin 10 µM | 17 | 2.2 |  | 2 |
|  | FSBA 1 mM | 14 | 1.9 |  | 2 |
| Y307C/V982C | Basal | 9 | 1.0 | 2.6 | 13 |
|  | Verapamil 50 µM | 28 | 3.0 | 2.6 | 3 |
|  | QZ59-*SSS* 1 µM | 29 | 3.2 |  | 2 |
|  | Valinomycin 10 µM | 33 | 3.5 | 3.9 | 4 |
|  | FSBA 1 mM | 20 | 2.1 |  | 2 |
| V982C | Basal | 11 | 1.0 | 2.2 | 9 |
|  | Verapamil 50 µM | 25 | 2.2 | 1.5 | 3 |
|  | QZ59-*SSS* 1 µM | 23 | 2.1 |  | 2 |
|  | Valinomycin 10 µM | 13 | --- |  | 2 |
|  | FSBA 1 mM | 28 | 2.4 |  | 2 |
| F343C/V982C | Basal | 18 |  | 2.7 | 9 |
|  | Verapamil 50 µM | 74 | 4.1 | 1.0 | 3 |
|  | QZ59-*SSS* 1 µM | 67 | 3.7 |  | 2 |
|  | Valinomycin 10 µM | 56 | 3.1 |  | 2 |
|  | FSBA 1 mM | 50 | 2.7 |  | 2 |
| F343C | Basal | 19 |  | 3.3 | 7 |
|  | Verapamil 50 µM | 46 | 2.5 | 1.5 | 3 |
|  | QZ59-*SSS* 1 µM | 42 | 2.2 |  | 2 |
|  | FSBA 1 mM | 39 | 2.1 |  | 2 |
| F728C | Basal | 6 | 1.0 | 2.0 | 9 |
|  | Verapamil 50 µM | 18 | 3.0 |  | 2 |
|  | QZ59-*SSS* 1 µM | 13 | 2.2 |  | 2 |
|  | Valinomycin 10 µM | 23 | 3.9 |  | 2 |
|  | FSBA 1 mM | 11 | 1.8 |  | 2 |
| F728C/V982C | Basal | 4 | 1.0 | 1.6 | 14 |
|  | Verapamil 50 µM | 9 | 2.3 |  | 2 |
|  | QZ59-*SSS* 1 µM | 10 | 2.5 |  | 2 |
|  | Valinomycin 10 µM | 10 | 2.5 | 2.0 | 4 |
|  | FSBA 1 mM | 5 | 1.1 |  | 2 |
| F978C | Basal | 12 | 1.0 | 1.4 | 5 |
|  | Verapamil 50 µM | 57 | 4.6 |  | 2 |
|  | QZ59-*SSS* 1 µM | 46 | 3.8 |  | 2 |
|  | Valinomycin 10 µM | 18 | 1.5 |  | 2 |
| Y307C/Q725C/V982C | Basal | 5 | 1.0 | 1.5 | 10 |
|  | Verapamil 50 µM | 12 | 2.6 |  | 2 |
|  | QZ59-*SSS* 1 µM | 14 | 2.8 |  | 2 |
|  | Valinomycin 10 µM | 14 | 2.8 | 2.1 | 4 |
|  | FSBA 1 mM | 6 | --- |  | 2 |
