# Supplementary material for: Multiple Transport-Active Binding Sites Are Available for a Single Substrate on Human P-Glycoprotein (ABCB1)
Source: PLoS One. 2013 Dec 5;8(12):e82463. doi: 10.1371/journal.pone.0082463 (PMC3857843; doi:10.1371/journal.pone.0082463)
Supplement: Table S3 — Effect of cyclosporine A and tariquidar on verapamil-stimulated ATPase activity of mutant Pgps. Inhibition of verapamil-stimulated ATP hydrolysis by CsA (10 µM) and tariquidar (5 µM) are shown. The ATPase activity is measured in the presence of verapamil (5 µM) because of the low basal activity of most of the mutants. ver, verapamil. (DOC) [file pone.0082463.s009.doc]

| **Table S3. Effect of cyclosporine A and tariquidar on verapamil-stimulated ATPase activity of mutant Pgps** | | | | | |
| --- | --- | --- | --- | --- | --- |
| Pgp mutant | Drug | ATP hydrolysis  (nmoles P_i_/min/mg protein) | | | |
|  |  | Average | %-inhibition | STDEV | # expts |
| Cysless WT | verapamil 5 µM | 34 |  | 4.4 | 4 |
| (control) | ver + CsA 10 µM | 15 | 58 |  | 2 |
|  | ver + tariquidar 5 µM | 5 | 84 |  | 2 |
| Q725C | verapamil 5 µM | 11 |  | 0.4 | 5 |
|  | ver + CsA 10 µM | 7 | 32 | 0.9 | 3 |
|  | ver + tariquidar 5 µM | 5 | 50 |  | 2 |
| Q725C/V982C | verapamil 5 µM | 8 |  | 4.5 | 12 |
|  | ver + CsA 10 µM | 8 | 4 | 1.6 | 4 |
|  | ver + tariquidar 5 µM | 2 | 75 | 2.2 | 8 |
| Y307C | verapamil 5 µM | 12 |  | 1.1 | 5 |
|  | ver + CsA 10 µM | 7 | 40 | 0.3 | 3 |
|  | ver + tariquidar 5 µM | 6 | 52 |  | 2 |
| Y307C/V982C | verapamil 5 µM | 14 |  | 10.5 | 3 |
|  | ver + CsA 10 µM | 7 | 52 |  | 1 |
|  | ver + tariquidar 5 µM | 1 | 91 |  | 2 |
| V982C | verapamil 5 µM | 26 |  | 1.3 | 4 |
|  | ver + CsA 10 µM | 10 | 61 |  | 2 |
|  | ver + tariquidar 5 µM | 6 | 76 |  | 2 |
| F728C | verapamil 5 µM | 16 |  | 2.0 | 5 |
|  | ver + CsA 10 µM | 10 | 36 | 0.7 | 3 |
|  | ver + tariquidar 5 µM | 7 | 58 |  | 2 |
| F728C/V982C | verapamil 5 µM | 8 |  | 1.3 | 5 |
|  | ver + CsA 10 µM | 1 | 81 |  | 2 |
|  | ver + tariquidar 5 µM | 5 | 34 | 1.7 | 3 |
| F978C | verapamil 5 µM | 33 |  | 4.0 | 4 |
|  | ver + CsA 10 µM | 18 | 45 |  | 2 |
|  | ver + tariquidar 5 µM | 26 | 21 |  | 2 |
| Y307C/Q725C/V982C | verapamil 5 µM | 12 |  | 0.5 | 4 |
|  | ver + CsA 10 µM | 6 | 46 |  | 2 |
|  | ver + tariquidar 5 µM | 5 | 56 |  | 2 |
