# Supplementary material for: Multiple Transport-Active Binding Sites Are Available for a Single Substrate on Human P-Glycoprotein (ABCB1)
Source: PLoS One. 2013 Dec 5;8(12):e82463. doi: 10.1371/journal.pone.0082463 (PMC3857843; doi:10.1371/journal.pone.0082463)
Supplement: Tables S4 — AutoDock Vina docking of cyclosporine A, tariquidar, valinomycin and FSBA in the homology model of human Pgp. Predicted modes with their respective docking scores are reported. A flexible receptor grid centered at the position of the bound-inhibitor QZ59-RRR (x=19.317, y=52.588 and z=-0.676) and an inner box of dimensions 40Å x 35Å x 35Å, was used to search binding poses of the ligands. The flexible receptor was defined with 26 residues of the binding pocket (see methods section). (DOC) [file pone.0082463.s010.doc]

| **Tables S4.** **AutoDock Vina docking of cyclosporine A, tariquidar, valinomycin and FSBA in the homology model of human Pgp** | | | | |
| --- | --- | --- | --- | --- |
| **Mode** | **Docking scores (kcal/mol)** | | | |
|  | **CsA** | **tariquidar** | **valinomycin** | **FSBA** |
| **1** | -9.7 | -12.9 | -10.8 | -9.9 |
| **2** | -9.6 | -12.7 | -10.8 | -9.8 |
| **3** | -9.5 | -12.4 | -10.6 | -9.7 |
| **4** | -9.3 | -12.3 | -10.4 | -9.6 |
| **5** | -9.3 | -12.2 | -10.4 | -9.6 |
| **6** | -9.2 | -12.0 | -10.4 | -9.4 |
| **7** | -9.1 | -12.0 | -10.3 | -9.4 |
| **8** | -9.1 | -11.9 | -10.2 | -9.4 |
| **9** | -9.0 | -11.8 | -10.2 | -9.3 |
| **10** | -9.0 | -11.8 | -10.1 | -9.1 |
